# Quantifying Uncertainty in Brain Age Predictions via Conformal Prediction

**DOI:** 10.64898/2026.09.04.749442

**Published:** 2026-09-11

**Authors:** Ujjwal Samanta, Golia Shafiei, Helmet Karim, Theodore D. Satterthwaite, Andrew Chen

## Abstract

Youth can exhibit signs of delayed or accelerated neurodevelopment, measurable via brain magnetic resonance imaging (MRI). Brain age prediction seeks to estimate brain age at an individual level using machine learning models fitted on healthy individuals. The brain age gap (BAG)–the difference between brain age and chronological age–has been studied as a potential biomarker. However, BAG suffers from known limitations including dependence on chronological age, regression to the mean, and challenges in interpretability. As an alternative framework, we introduce brain age intervals (BAIs) as a normative interval on an individual’s chronological age, based on MRI measures. BAIs can be derived using a recent statistical framework called conformal prediction that yields prediction intervals with guaranteed coverage. We train several brain age prediction models on structural and functional MRI scans from the Reproducible Brain Charts dataset (aged 6-22) and estimated BAIs. The BAIs demonstrated stable empirical coverage close to the nominal 90% level (median coverage=91%, width=7.36 years, root mean square error (RMSE)=2.20 years across 100 repeated train–test splits). Association between interval coverage and clinical measures (p-factor, parental education) were weak, with only a modest directional signal for parental education. Together, these findings demonstrate the feasibility of uncertainty-aware brain age modeling in youth populations while highlighting the need for larger and more diverse samples to detect meaningful clinical and environmental associations.

## Full Text Availability

The license terms selected by the author(s) for this preprint version do not permit archiving in PMC. The full text is available from the preprint server.

